# Osteoma involving the olfactory groove: evaluation of the risk of a CSF leak during endoscopic surgery

**DOI:** 10.1007/s00405-020-05938-4

**Published:** 2020-04-06

**Authors:** Tomasz Gotlib, Magdalena Kuźmińska, Paulina Kołodziejczyk, Kazimierz Niemczyk

**Affiliations:** Department of Otorhinolaryngology Head and Neck Surgery, Medical University of Warsaw, ul. Banacha 1a, 02-097 Warsaw, Poland

**Keywords:** Olfactory groove, Olfactory fissure, Olfactory cleft, Lateral lamella, Complications, Cerebrospinal fluid leak, Endoscopic sinus surgery, Endoscopic skull base surgery, Osteoma

## Abstract

**Purpose:**

The olfactory groove (OG) is a common site of iatrogenic cerebrospinal fluid (CSF) leak during endoscopic sinus surgery. We aimed to evaluate the prevalence of CSF leak during endoscopic removal of osteomas involving the OG and identify CT findings indicating increased risk of this complication.

**Methods:**

A retrospective review was conducted of patients operated on for frontoethmoidal osteoma from 11 years in a single institution. A retrospective review of the literature, 1999 to 2019, of perioperative complications in patients operated on for frontoethmoidal osteoma using endoscopic or combined approaches.

**Results:**

Case series: 73 patients were identified including 17 with the OG involvement. The only case of CSF leak occurred in a patient with spongious part of osteoma at the OG. Among six osteomas with spongious component at the OG, one was detached and five had to be drilled down, leaving a small remnant in four. In contrast, all the 11 osteomas with ivory part at the OG were safely detached and completely removed from the OG after debulking. The prevalence of CSF leak was not statistically different between the patients without and with involvement of the OG. Systematic review of the literature: Among the 273 identified patients there were 8 cases of intraoperative CSF leaks (3%) including 2 from the OG (0.7%).

**Conclusion:**

Involvement of the OG does not significantly increase the risk of intraoperative CSF leak. However, this risk may be increased in patients with the spongious part of the tumor attached to the OG.

## Introduction

Osteoma is a benign, slow-growing tumor, rarely requiring surgical treatment. The most common location within the paranasal sinuses is the frontoethmoidal junction [1]. Indications to treatment include fast growth, drainage blockage, frontal pain/headache and extension beyond the sinus borders [2, 3].

Transnasal endoscopic management of frontal sinus osteomas has become feasible and safe due to technological development and growing surgical experience [4, 5].

The need for external approach has been decreasing over the past decade. Endoscopic approach to large osteomas can be more time-consuming compared to open approaches due to the amount of bone that has to be removed through a narrow corridor.

This disadvantage can be overcome to some extent by the use of high-speed curved drills (30.000 RPM) that were introduced recently. Involvement of the OG is not treated as contraindication to endoscopic approach; however, it may present a challenge [2]. Unlike during standard FESS procedures, in these cases it is difficult to avoid direct contact of surgical tools or pressure on the lateral lamella of the cribriform plate, which is known to be the most vulnerable part of the skull base. On the preoperative CT, osteoma often seems to fuse with the lateral lamella of the cribriform plate. Most published series of patients undergoing endoscopic removal of osteomas are relatively small and do not provide information on how many tumors were located at the OG, and if there were any intraoperative difficulties related to this location of osteoma [3, 5, 6].

The aim of this study is to evaluate the prevalence of CSF leak during endoscopic removal of osteomas involving the OG and identify CT findings indicating increased risk of this complication.

## Material and methods

### Case series

We carried out retrospective review of the patients operated on using transnasal endoscopic approach for frontal sinus and recess osteoma by the first author (TG) from January 2008 to December 2018. Data on intraoperative complications were analyzed. To assess the risk of CSF leak, we compared data of patients with OG involvement and patients with involvement of the posterior wall of the frontal sinus or recess, with no tumor at the OG. The differences between the two groups were assessed using Fisher’s exact test. Preoperative CT scans of the patients with osteoma involving the OG were evaluated using multiplanar reconstruction using OsiriX (Pixemo, Switzerland) software in the bone window.

### Operative technique

Anterior ethmoidectomy was performed using standard endoscopic sinus surgical instruments and a 30° scope. If not occluded by the tumor, frontal ostium was widened with curved irrigated 70° and 15° diamond burrs and/or with Kerrison punches. Exposure of the tumor involving the OG on one side, with no massive extension into the frontal sinus, was accomplished by performing Draf IIa, or IIb. Draf IIb procedure was occasionally extended with removal of the upper part of the nasal septum, removal of intersinus septum or both above-mentioned structures. The drill was then used to debulk the tumor and reduce its anterior and lateral part. Mobilization was attempted by pulling the remnant away from the OG.

Large osteomas crossing the midline were removed using Draf III procedure. These tumors were at first drilled down in front of the OG together with inner part of the frontal beak. The lateral parts were drilled in posterior direction exposing the fovea ethmoidalis. Part of the tumor above the OG was removed, up to the level of the posterior table of the frontal sinus. Finally, the remnant was detached with a curette.

Angled 45° and 70° scopes were used to ensure completeness of removal. Routinely, no additional external approaches were used. Tumors that could not be detached after debulking were drilled down.

The schedule of postoperative follow-up included saline nasal douches for 3 weeks starting the day after surgery and intranasal steroids for up to 4 weeks starting 10 days postoperatively. The sinus cavity was cleaned every week for at least 1 month. Follow-up was performed by nasal endoscopy. Postoperative CT was performed in case of uncertainty regarding completeness of the removal or frontal ostium closure.

### Review of the literature

We performed a systematic review of the literature using PubMed, Embase and Scopus with the key words “sinus”, “osteoma” and “endoscopic surgery” with limitations to human subjects and the English language. Case series of five or more patients operated on for frontal/frontoethmoidal osteomas using endoscopic or combined endoscopic and external procedures published between 1999 and 2019 were retrieved. Articles that did not describe the relation between tumor location, surgical approach and complications were eliminated.

## Results

### Case series

Among 73 patients operated on there were 18 patients with the tumor involving the OG. One patient admitted to our hospital with CSF leak after unsuccessful surgery performed elsewhere was excluded from further evaluation. Data of the remaining 17 patients were evaluated further (Table 1).

**Table 1:** Overview of patients with osteoma at the OG

Patient no	Greatest dimension (cm)	Attachment to the OG horizontal × coronal (mm)	Spongious part at the OG	Crossing the midline anterior to the OG	Approach used	Remnant at the OG (mm)
1	4.8	12 × 5		1	Draf III	0
2	4.5	20 × 7		1	Draf III	0
3	3.6	16 × 8		1	Draf III	0
4	3.3	8 × 6		1	Draf III	0
5^a^	3.2	7 × 3	1	0	Extended Draf IIb	0
6	3.0	5 × 4	1	0	IIb	3 × 4 mm
7	2.8	12 × 4		1	Draf III	0
8	2.5	8 × 6	1	0	Draf IIb	0
9	2.3	4 × 2		1	Draf III	0
10	2.2	8 × 5	1	1	Draf III	3 × 7 mm
11	2.1	12 × 7	1	0	Draf IIa	3 × 8 mm
12	2.0	2 × 3		0	Draf III	0
13	2.0	2 × 1		0	Extended Draf IIb	0
14	1.7	9 × 4		0	Extended Draf IIb	0
15	1.0	8 × 5	1	0	Draf IIb	5 × 1 mm
16	1.0	4 × 2		0	Draf IIa	0
17	0.7	4 × 3		0	Draf IIa	0

^a^Patient with an intraoperative CSF leak

The most common indication for surgery was frontal pain (in seven patients), mucocele (9), orbital involvement (1), recurrent orbital emphysema (1), a history of meningitis episode in the patient with the posterior table destruction (1), and fast growth (1).

The tumor crossed the midline anterior to the OG in seven patients.

Three patients with giant osteomas underwent second stage procedure to remove the remnant located away from the OG. One of them, presenting with mucocele and destruction of the posterior table, required limited accessory external approach.

In all 17 cases, it was not possible to differentiate between adjacency and solid attachment of the tumor to the OG. Ten of these tumors were extending between  the anterior and posterior table of the frontal sinus. The mean greatest dimension of the tumor was 2.54 cm (range 0.7–4.8 cm).

There were five osteomas type 1, two—type 2, nine—type 3 and one—type 4 according to Chiu classification [6]. Assessment of the preoperative CTs with bony window adjustment revealed 11 mixed type osteomas, 4 exclusively spongious and 2 exclusively ivory.

**Fig. 1:**
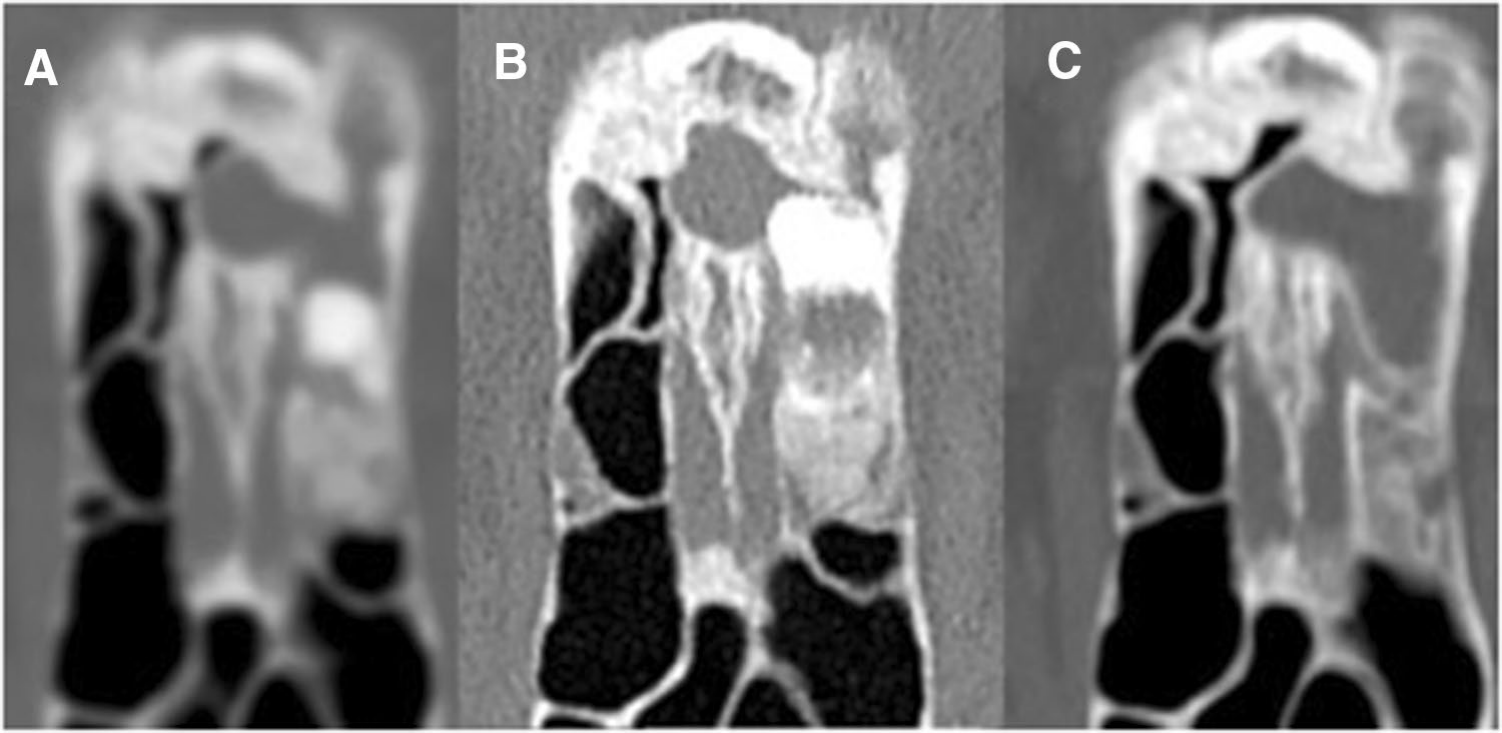
**a**, **b** Consecutive CT scans showing osteoma growth. **c** Postoperative scan showing opacification of the frontal sinus outflow tract

In one patient with mixed osteoma, consecutive CT examinations revealed tumor growth. The spongious part expanded, pushing the ivory component anteriorly (Fig. 1). Intraoperatively, the attachment was found in the spongious part. Similar centrifugal pattern with central spongious part and peripheral C- or U- shaped compact bone component was observed in the remaining ten mixed osteomas.

**Fig. 2:**
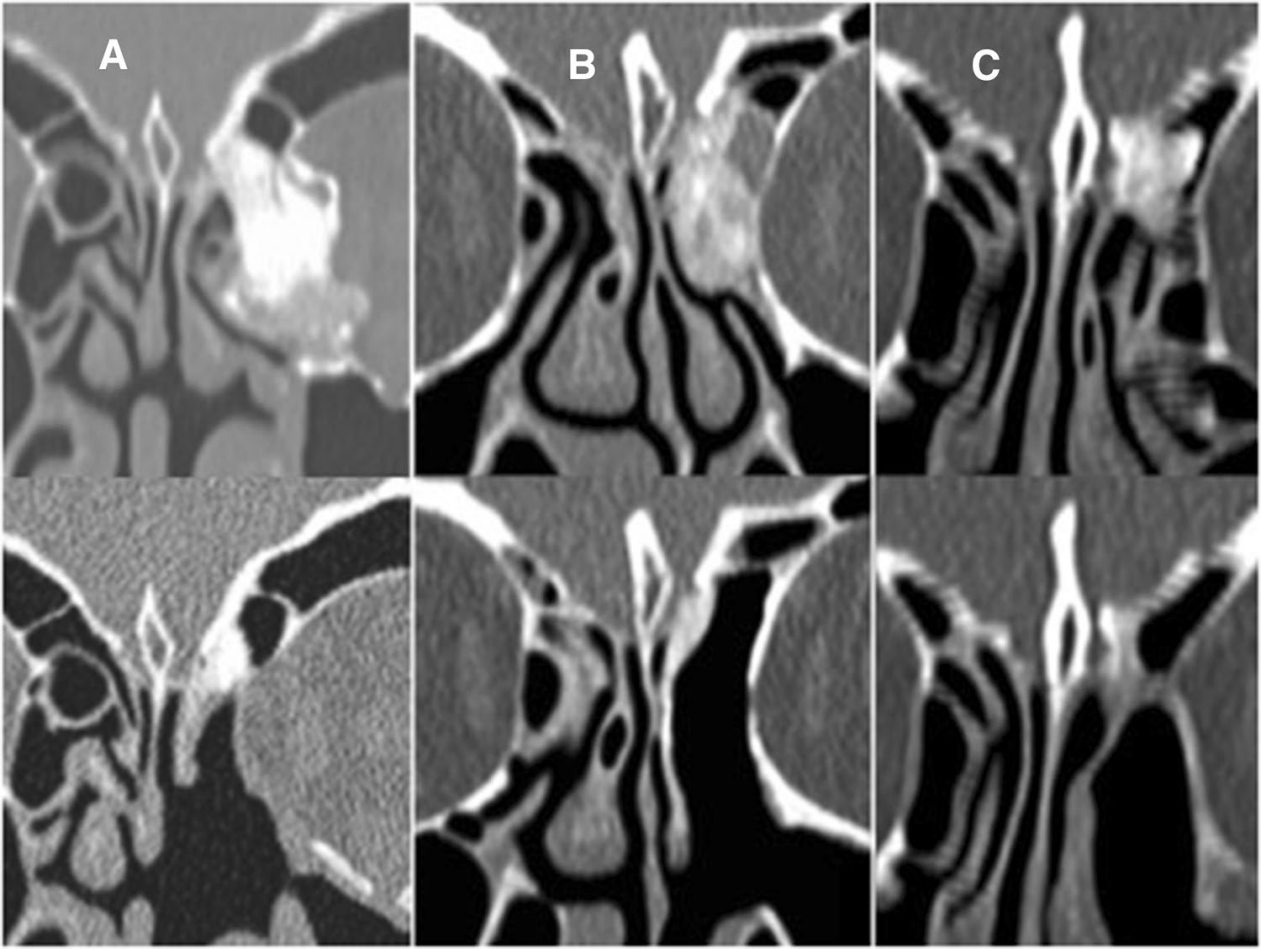
**a**–**c** CT of patients with osteomas with spongious component at the OG before and after surgery

The described surgical technique based on reduction of the tumor around the OG and detaching the remnant was not effective in five patients. In all of them, part of the tumor attached to the OG was composed of spongious bone and could be removed only with the drill (Fig. 2). In one of these patients, the remnant at the OG was completely removed but this resulted in CSF leak, which was successfully managed intraoperatively.

In the remaining four cases, part of the tumor at the OG was reduced and a small remnant was left. Overall, only one of six osteomas with the spongious part at the OG was successfully detached and completely removed without CSF leak.

**Fig. 3:**
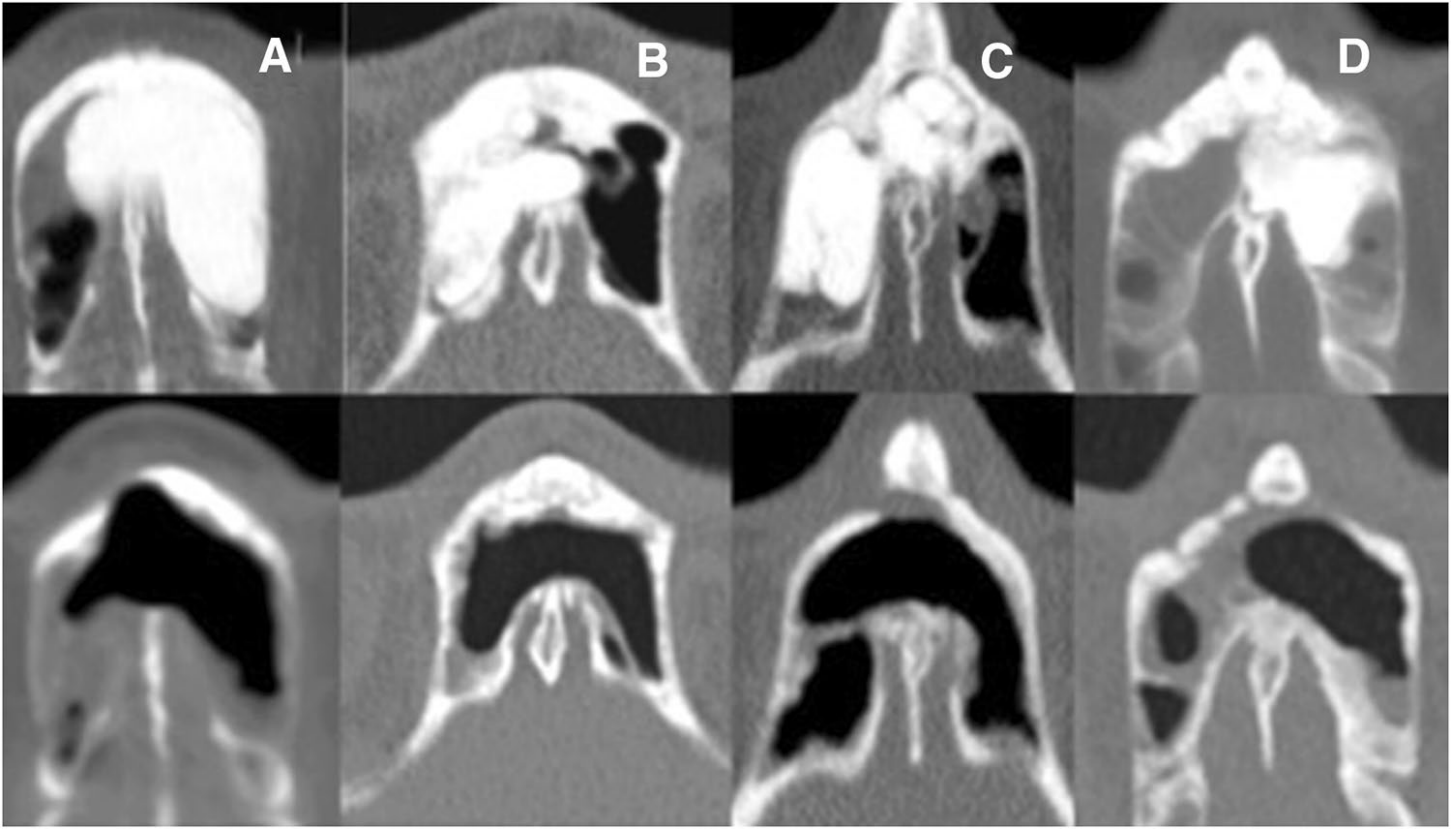
**a**–**d** CT axial views of patients with osteomas with compact component at the OG before and after surgery. Note the presence of postoperative osteoneogenesis in patient D

In contrast, all 11 osteomas with ivory remnant located at the OG were detached without complications (Fig. 3).

Among 35 patients with posterior table involvement with no tumor at the OG, there were  10 osteomas type 1, 24—type 3, and 1—type 4. Seventeen of these tumors extended between the anterior and posterior table of the frontal sinus. The mean greatest dimension of the tumor was 1.54 cm (range 0.6–3 cm).

Removal was incomplete in two patients (remnant greater than 10% of initial size). Two patients required reoperation due to closure of the frontal ostium.

There was no single case of CSF leak among 35 patients in this group. The difference in CSF leak prevalence between the two groups was not statistically significant (*p* > 0.05).

### Review of the literature

Full texts of the 18 identified studies were analyzed for perioperative complications and involvement of the OG by the tumor. Duplicate studies were eliminated. Data from the remaining 16 studies are summarized in Table 2. The number of patients with involvement of the OG was precisely described in only one study. In eight studies, involvement of the OG was mentioned cursorily, e.g., shown on CT scans. Among the patients operated on endoscopically, there were two cases of CSF leak that resulted from removal of osteoma together with the lateral lamella of the OG. In another two cases, the dura of the OG was exposed without CSF leak. There was no CSF leak in the combined approach group; however in one patient operated on endoscopically, the procedure was converted to combined after CSF leak occurred.

**Table 2:** Case series of more than five patients operated on using endoscopic or combined surgery (1999–2019)

Study	Endonasal	Combined/auxillary external approach	CSF leak	OG involvement	Other perioperative complications	Remnant
Brodish [7]	9	0	2, including 1 from the OG	1/npr	0	0
Schick^a^ [8]	12	0	3	npr	0	1, 2 re
Chiu [6]	3	5	0	1/npr	0	0
Dubin [9]	7	1	0	nr	0	1
Turri-Zanoni^b^ [4]	31	25	0	2/npr	Dura exposure at the OG 2 (E), diplopia (C)	2
Seiberling [5]	10	13	0	nr	0	4
Ledderose [3]	8	12	0	npr	Bleeding 2	0/npr
Ooi [10]	9	0	0	2/npr	0	0/npr
Pagella [11]	20	7	0	3/npr	0	0
Sieśkiewicz [12]	8	0	0	nr	Dura exposure 1	0
Celenk [13]	6	1	0	nr	Orbital hematoma 1	1
Gotlib [14]	29	0	0	7	0	3
Karligkiotis [15]	9	0	1 from the OG	1/npr	0	1, 1 re
Lee [1]	4	1	0	1/npr	0	0
Arslan [16]	11	5	0	nr	0	0
Wolf [17]	17	10	2 (1 E npr + 1 E converted to C)	npr	Bleeding, strabismus/npr	0
Total	193	80	8	–	10	13

*nr *not reported, *npr* not precisely reported, *C* combined endonasal and external approach, *E* purely endoscopic endonasal approach, *r*e removal of the remnant during another operation

^a^Microendoscopic endonasal surgery

^b^Duplicated data from two previous studies from the same institution (Bignami [18] and Castelnuovo [19]) were eliminated

The overall incidence of CSF leak for purely endoscopic approach was 4% (8/193) and 3% (8/273) for the whole group (endoscopic + combined). The incidence of the CSF leaks from the OG was 1% (2/193) and 0.7% (2/273), respectively. Other perioperative complications included bleeding (3 cases), orbital hematoma (2), persistent diplopia (2), and exposure of the dura in other locations than the OG (1).

## Discussion

The possibilities of endoscopic surgery of frontoethmoidal osteomas have expanded since the first report on transnasal removal of frontal sinus osteoma augmented with minitrephination published in 1992 [20]. Currently, tumors located lateral to the vertical plane through the lamina papyracea, attached to the anterior table of the frontal sinus, completely filling the frontal recess or ostium, or extending more than 2 cm beyond the level of the frontal ostium can be safely removed using a purely endoscopic approach. However, in patients with narrow frontal ostium, tumors widely attaching to the orbital roof or filling narrow supraorbital recess complete removal can be restricted [4, 5, 14].

The thickness of the lateral lamella of the OG is often below the resolution of CT. To our knowledge, there is no imaging modality that can reliably differentiate firm attachment from loose adherence of the tumor to the lateral lamella of the OG. Nevertheless, during the surgery intact mucoperiosteum covering the OG can be often visualized after detachment of osteoma. The site of attachment of small osteomas can be usually found on CT; however in large tumors it is difficult to find it preoperatively as well as during the procedure [4]. The most accepted theory on osteoma growth presumes that it grows from a well-vascularized central fibrous part containing osteoblasts. The peripheral part, composed of ivory bone, does not grow. This theory justifies leaving the peripheral ivory remnant if it is impossible to remove it [5]. This also implies that the ivory part of mixed osteoma should not be attached to the OG even if there is no visible gap between the lateral lamella of the OG and osteoma on CT. Our findings seem to support this hypothesis. On the other hand, the attachment built from spongious bone may mature and become ivory over time. We observed this type of ivory attachment on the posterior wall of the frontal sinus in some patients, but never at the OG.

If the tumor is firmly attached to the OG, it must be drilled down. Skeletonizing the OG surrounded by osteoma with the drill is time-consuming and poses high risk of CSF leak.

During the procedure, we tried to keep the mucosa intact whenever possible; however, it was not always feasible. Removal of mucoperiosteum and leaving denuded bone induce neo-osteogenesis. This neo-osteogenesis is impossible to distinguish from small remnant of osteoma on late postoperative CT (Fig. 3d).

Cavitation technique is a mainstay of surgical management for fibroosseous tumors [18]. In case of large osteoma involving the OG on both sides, pulling the remnant up creates pressure against the posterior table, which is more resistant than the lateral lamella of the OG. Similarly, in case of smaller osteomas (e.g., wedged between the lateral lamella of OG and lamina papyracea), pulling the remnant in the lateral and anterior direction prevents fractures of the lateral lamella. Uncontrolled, forced maneuvers without prior diminishing the size of the tumor effected in CSF leak in the patient with ivory osteoma referred to our institution. During the revision procedure, we reduced the tumor with the drill, removed the remnant and sealed the fracture of the lateral lamella with the dura substitute. This patient was excluded from further evaluation to ensure the homogeneity of the group.

The limitation of our literature review is that data on tumor attachment, consistency and details of surgical procedures could not be sufficiently linked together. Studies published before 1999 were not analyzed because surgical experience with endoscopic removal of osteomas was very limited at that time. There are two recently published systematic reviews on surgery of frontal sinus osteomas [21, 22]. One of them focuses on postoperative complications and does not analyze the site of CSF leaks. Complications occurred in 7.5% of endoscopic and 8.8% of combined cases analyzed in this study. These values are slightly higher compared to our findings. The surgical skills of authors reporting case series are likely to be better than those reporting single cases. Eliminating case reports could potentially bias the results and lower the rate of complications in our study. The true complication rate may be higher, as many complications remain unreported.

## Conclusions

Involvement of the OG does not significantly increase the risk of CSF leak during osteoma removal. Reducing the tumor around the olfactory grove before its final detachment decreases the risk of CSF leak. However, the spongious part of the tumor at the OG is likely to be firmly attached. In these cases, the best option is to carefully reduce the remnant with a drill.
